# Antennal sensilla in an anophthalmic wood‐dwelling species, *Clinidium canaliculatum*, Costa 1839 (Coleoptera, Rhysodidae)

**DOI:** 10.1002/jemt.23969

**Published:** 2021-10-22

**Authors:** Anita Giglio, Antonio Mazzei, Maria Luigia Vommaro, Pietro Brandmayr

**Affiliations:** ^1^ Department of Biology, Ecology and Earth Science University of Calabria Arcavacata di Rende Italy; ^2^ Natural History Museum and Botanical Garden University of Calabria Arcavacata di Rende Italy

**Keywords:** chemoreceptor, hygroreceptor, mechanoreceptor, mycophagy, saproxylic

## Abstract

The habit of feeding on slime moulds (Myxomycetes) commonly present in litter or dead wood requires specific morphological adaptations of the mouthparts and sensory structures involved in the search for habitat and food. In this study, the external morphology of antenna and its sensilla were studied using scanning electron microscopy in the saproxylic beetle, *Clinidium canaliculatum*, Costa 1839 (Coleoptera, Rhysodidae). Their moniliform antennae consist of a scape, pedicel, and nine flagellomeres. We identified seven different types of sensilla, according to their morphological characteristics: two types of sensilla chaetica (sc1 and 2), two types of sensilla basiconica (sb1 and 2), one type of sensilla campaniformia, one type of sensilla coeloconica, and Böhm sensilla. No sexual dimorphism was found regarding antennal morphology and sensilla type and distribution, except for the sensilla coeloconica. The functional role of these sensilla was discussed in relation to their external structure and distribution, and compared with the current knowledge on coleopteran sense organs. Results are basic information for further physiological and behavioral studies to identify their role in the selection of habitat, food, mates and oviposition sites.

## Graphical abstract

- Scanning electron microscopy was used to investigate the sensorial equipment of a saproxylic species feeding on slime moulds, *Clinidium canaliculatum*
- Six types of sensilla were described on the moniliform antennae of both sexes, including two types of sensilla chaetica, two types of sensilla basiconica, one type of sensilla campaniformia, and one type of sensilla coeloconica
- The distribution of sensilla suggested that the antennae are involved in the scanning surrounding area for habitat selection and in feeding behavior to compensate for the absence of eyes

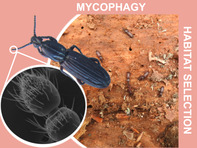

## 1 INTRODUCTION

Saproxylic insects are a functional group of species that play a key role in forest ecosystems and include bark‐feeders, feeders on wood‐decomposing fungi, associated predators, parasitoids, detritivores, and other commensals (Bouget, Larrieu, & Brin, 2014; Jonsell & Weslien, 2003; Stokland, Siitonen, & Jonsson, 2012). *Clinidium canaliculatum*, Costa 1839 (Coleoptera, Rhysodidae) is an obligate saproxylic species inhabiting montain forests in central and southern Italy and Greece (García et al., 2019; Mazzei, Audisio, Taglianti, & Brandmayr, 2019). As larvae and adults live in rotten wood of conifers and feed on Myxomycetes (Bell, 1994; Hammond & Lawrence, 1989), they are an important component of the biodiversity in old‐growth forest communities. This species has been listed as vulnerable in the red list of the International Union for Conservation of Nature (IUCN) because it is susceptible to intensive forest management that reduces the abundance of dead wood (Carpaneto et al., 2015).

Insect antennae are highly developed, paired, segmented sensory appendages with multimodal functions covered by different sensilla involved in chemo‐, hygro‐, thermo‐, and mechanoreception (Altner & Prillinger, 1980; Schneider, 1964; Zacharuk, 1985). Antennal shape and sensilla distribution are the result of environmental selective pressure to increase the efficiency of detecting physical and chemical signals that are crucial in the search for habitat, prey, host, partner, and oviposition site (Elgar et al., 2018). Antennal morphology and ultrastructure of sensilla have been reported in several families of Coleoptera, such as Anobiidae (Abd El‐Ghany & Abd El‐Aziz, 2021), Bruchidae (Wang, Zheng, Zhang, & Zhang, 2018), Cerambycidae (Di Palma, Pistillo, Griffo, Garonna, & Germinara, 2019; Dong et al., 2020; Faucheux, 2011), Carabidae (Di Giulio, Maurizi, Rossi Stacconi, & Romani, 2012; Giglio et al., 2008; Merivee et al., 2002; Ploomi et al., 2003), Coccinellidae (Hao, Sun, & Liu, 2020; Sevarika, Rondoni, Conti, & Romani, 2021), Curculionidae (Chen, Zhang, Wang, & Kong, 2010; Romani et al., 2019; Shi et al., 2021), Elateridae (Faucheux, Németh, Hoffmannova, et al., 2020), Scarabaeidae (Shao, Sun, Wang, & Chen, 2019; Zauli et al., 2016a, 2016b), Tenebrionidae (Faucheux, 2013; Seada & Hamza, 2018), and Buprestidae (Faucheux, Németh, Hoffmannova, et al., 2020). However, there is no information on the sensorial pattern of species belonging to Rhysodidae, except for taxonomic studies on the distribution of setae in larval stages (Bousquet & Goulet, 1984; Makarov, 2008).

This study was designed to describe the antennal sensory equipment of females and males in *C. canaliculatum*. Shape, size, number, and distribution of the sensilla were studied using scanning electron microscopy. The function of the sensilla found in *C. canalicutatum* was discussed comparatively with current knowledge on coleopteran sense organs. This research provides basic information for further ultrastructural, behavioral, and ecological studies.

## 2 MATERIALS AND METHODS

### 2.1 Insect collection

**FIGURE 1:**
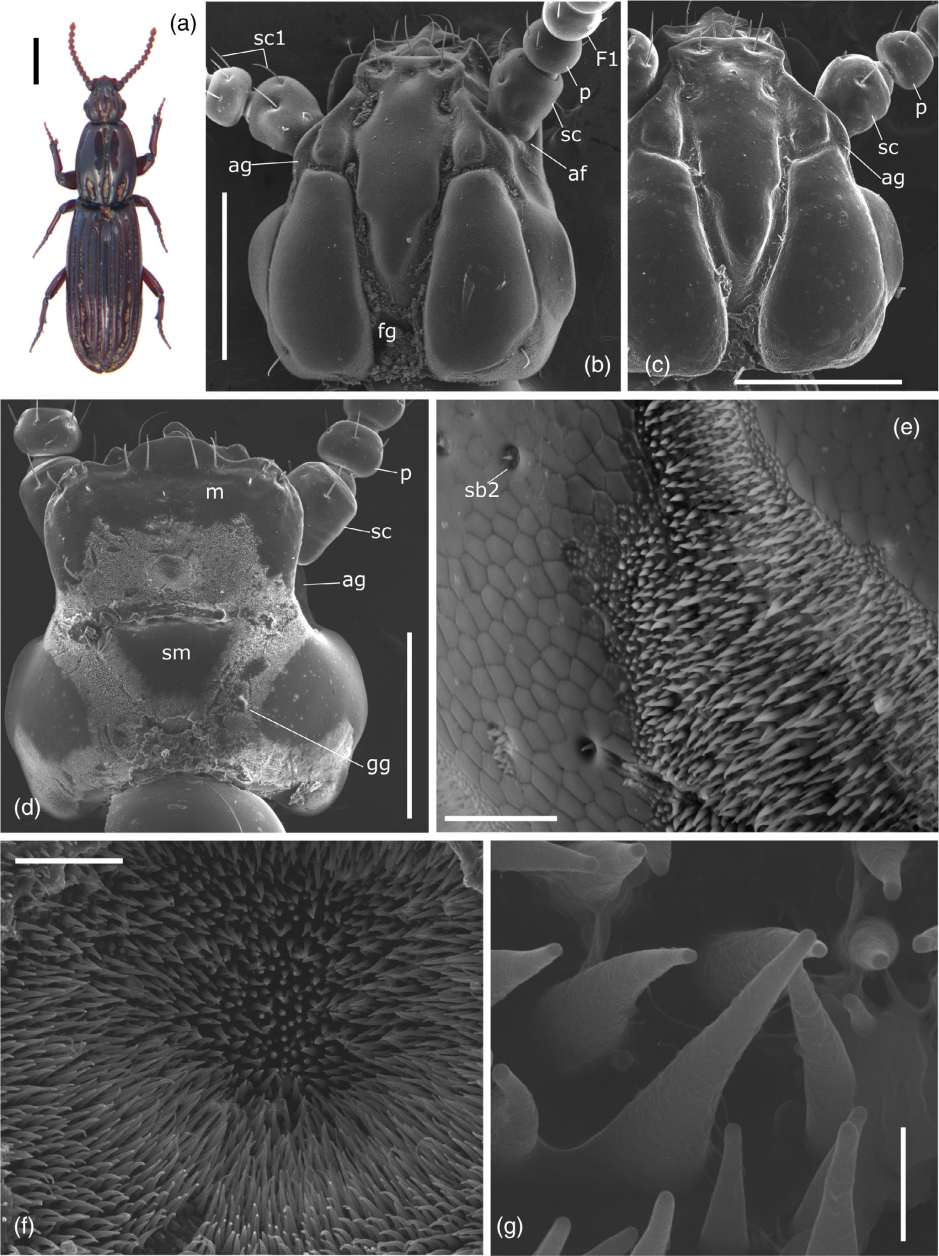
(a) *Clinidium canaliculatum* female. (b,c) Head dorsal view of male (b) and female (c). (d) Head ventral view, female. (e) Magnification of sensorial area on the antennal groove (ag). (f) Magnification of sensorial area on the mentum (m). (g) Detail of sensilla in (f). ag: antennal groove; af: antennal foramen; fg: frontal groves; F1: first flagellomere; gg: gular grooves; p: pedicel; sb2: sensilla basiconica type 2; sc: scape; sc1: sensilla chaetica type 1; sm: submentum. Bars: 2 μm (g); 20 μm (e,f); 500 μm (b–d); 1 mm (a)

Adults of *C. canaliculatum* (Figure 1a) were hand‐collected under rotten pine bark in the Sila National Park (39°21′16.79″N, 16°37′57.64″E, Monte Spina 1,550 m a.s.l. San Giovanni in Fiore, Calabria, Southern Italy) in May 2021. In the laboratory, beetles were identified by using a dichotomous key and separate by gender.

### 2.2 Scanning electron microscopy

Beetles (three males and three females) were anesthetized in a cold chamber at 4°C for 3 min and beheaded under a stereomicroscope. Heads were fixed in 2.5% glutaraldehyde and 1% paraformaldehyde in phosphate buffer (PBS, 10 mM pH 7.4; Electron Microscopy Sciences) over night at 4°C. They were then washed with PBS (Sigma‐Aldrich), dehydrated in an ethanol (Sigma‐Aldrich) series and finally specimens were immersed in hexamethyldisilazane (HMDS, (Sigma‐Aldrich) to remove liquids. Heads of males and females were mounted on aluminum stubs with double‐sided sticky tapes with ventral or dorsal sides. Immediately before the observation, they were graphite coated in a Sputter ‐ Carbon Coater (QUORUM Q150T‐ES). Specimens were examined by a scanning electron microscope (Electron Probe Micro Analyzer [EPMA]—JEOL‐JXA 8230; Microscopy and Microanalysis Centre, CM2,—University of Calabria, Italy) operating at an accelerating voltage of 15 kV.

### 2.3 Data analyses

Measurements were taken with ImageJ open source software on digitized images and processed as means ± standard error. The morphofunctional types of the sensilla were described and classified according to Schneider (1964) and Zacharuk (1985).

The differences between the sexes for both antenna and numbers of sensilla were assessed by the nonparametric Mann–Whitney *U* Test. Statistical analyses were performed using R version 3.0.1 software (R Development Core Team 2013).

## 3 RESULTS

### 3.1 Gross morphology

**FIGURE 2:**
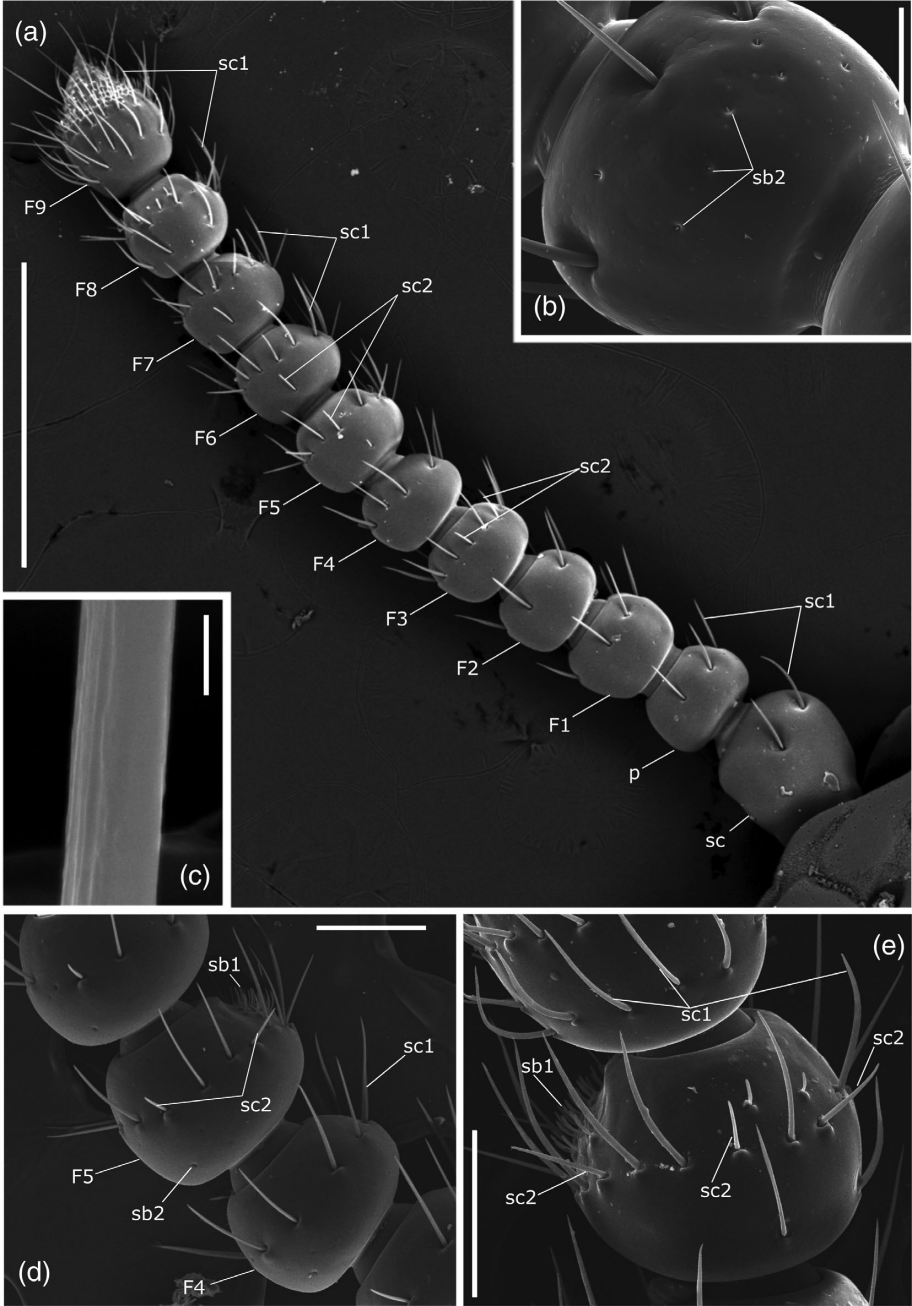
*Clinidium canaliculatum* antennae. (a) Dorsal view of antenna, male. (b) Pedicel, male. (c) Detail of sensillum chaeticum type 1. (d) Lateral view of fourth and fifth flagellomere, male. (e) latero‐dorsal view of sixth and seventh flagellomere, female. F1–9: flagellomeres; p: pedicel; sb1: sensilla basiconica type 1; sb2: sensilla basiconica type 2; sc: scape; sc1: sensilla chaetica type 1; sc2: sensilla chaetica type 2. Bars: 2 μm (c); 50 μm (b); 100 μm (d,e); 500 μm (a)

The moniliform antennae of *C. canaliculatum* were composed of three segments: scape, pedicel, and flagellum (Figures 1b–d and 2a). Each antenna had a total length of 1.95 ± 0.02 mm (*n* = 3) in females and 1.70 ± 0.03 mm (*n* = 3) in males. No significant differences were found in the shape and length of the antennae between sexes (Table 1; *p* >.05). The scape was the first elongated segment, articulated basally on the antennal foramen by a globular condyle (Figures 1b–d and 2a). The pedicel, directly connected to the scape, was spherical in shape (Figure 2a,b). The flagellum was formed by nine flagellomeres (F1–F9; Figure 2a) and had a total length of 1.38 ± 0.018 mm in females and 1.26 ± 0.024 mm in males (Figure 2a; Table 1). All flagellomeres, except the last one, were spherical in shape. The compound eyes were absent (Figure 1b–d).

**TABLE 1:** Length (μm) of antennal segments in males and females of *C. canaliculatum*

Sex	Scape	Pedicel	Flagellomeres								
			1	2	3	4	5	6	7	8	9
**♂**	226.92 ± 22.88	135.75 ± 22.88	122.62 ± 6.17	116.20 ± 1.05	125.90 ± 2.80	121.22 ± 6.44	131.68 ± 8.42	130.65 ± 3.51	136.05 ± 8.07	144.22 ± 3.81	237.63 ± 2.31
**♀**	347.66 ± 16.47	152.20 ± 5.46	139.61 ± 3.80	133.78 ± 3.36	133.05 ± 3.49	129.56 ± 3.08	144.04 ± 3.80	141.98 ± 4.93	145.29 ± 8.01	152.29 ± 0.93	259.13 ± 3.91

*Note*: Data are presented as mean ± *SE*, (*n* = 3). Data are not significantly different between sexes (Mann–Whitney *U* test, *p* >.05.

**FIGURE 3:**
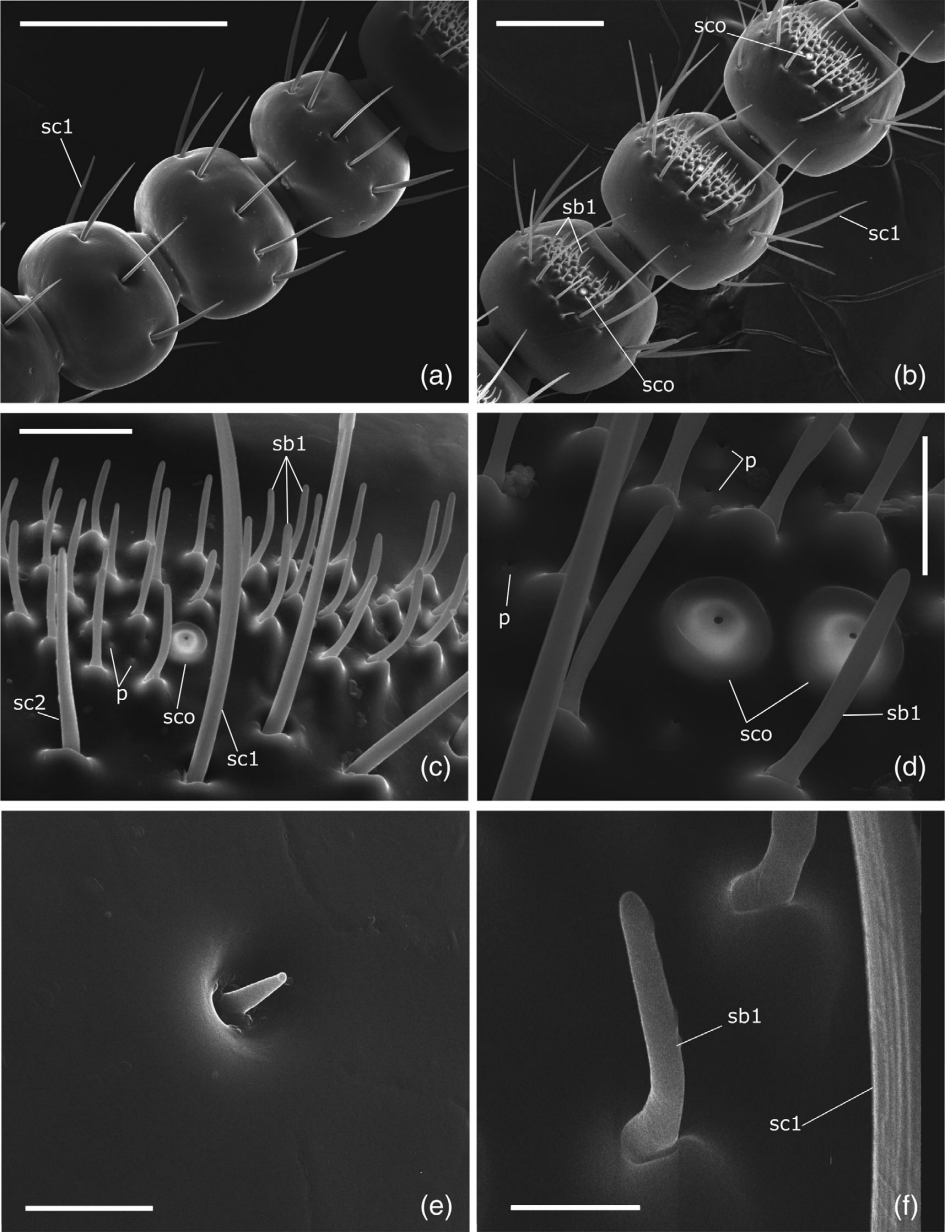
*Clinidium canaliculatum* antennae. (a) Ventral view of male antenna, second, third, fourth flagellomere. (b) Ventral view of fifth to eighth flagellomere, male. (c) Sensorial area on the ventral side of seventh flagellomere, male. (d) Magnification of sensorial area on the ventral side of seventh flagellomere, female. (e) Magnification of sensillum basiconicum type 2 on pedicel. (f) Detail of sensillum basiconicum type 1. p: glandular pore; sb1: sensilla basiconica type 1; sc1: sensilla chaetica type 1; sc2: sensilla chaetica type 2; sco: sensilla coeloconica. Bars: 5 μm (e,f); 10 μm (d); 20 μm (c); 100 μm (b); 200 μm (a)

**FIGURE 4:**
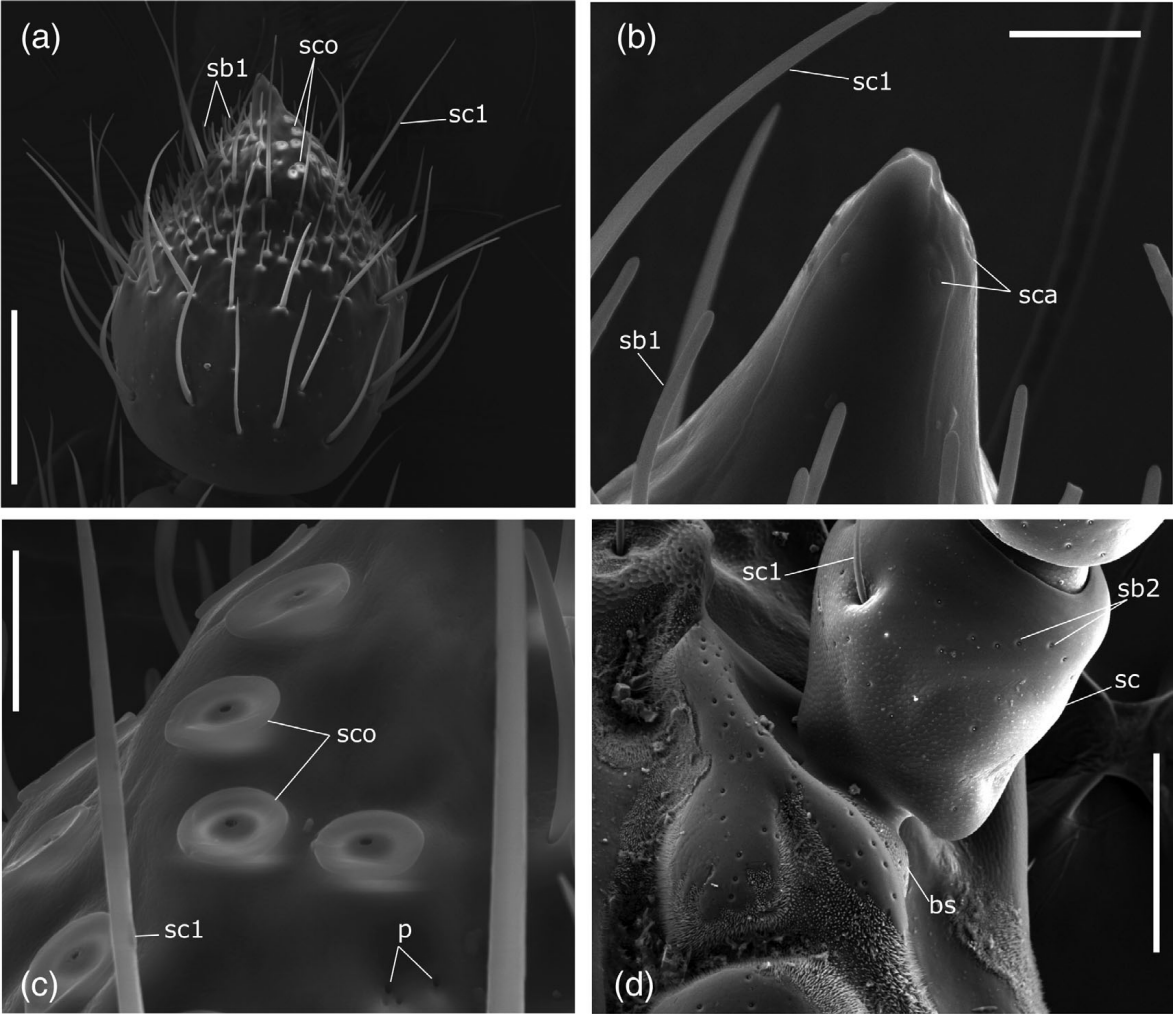
*Clinidium canaliculatum* antennae. (a) Dorsal view of ninth flagellomere, male. (b) Detail of sensilla campaniformia (sca) on the tip of ninth flagellomere. (c) Detail of sensilla coeloconica (sco) on the wall of ninth flagellomere. (d) Böhm sensilla (bs) on the condyle of the scape (sc). p: glandular pore; sb1: sensilla basiconica type 1; sb2: sensilla basiconica type 2; sc1: sensilla chaetica type 1. Bars: 10 μm (b, c); 100 μm (a); 150 μm (d)

Seven distinct morphological types of sensilla were identified according to their size and shape (Figures 2, 3, 4): sensilla chaetica type 1, sensilla chaetica type 2, sensilla basiconica type 1, sensilla basiconica type 2, sensilla coeloconica, sensilla campaniformia, and Böhm sensilla. There were no consistent gender differences in the type, topography, or number of sensilla. Moreover, we also identified large areas of sensilla dorsally on the frontal and antennal grooves and ventrally on the mentum and gular grooves (Figure 1b–g).

#### 3.1.1 Sensilla chaetica

Sensilla chaetica type 1 (sc1) were bristles (95 ± 2.15 μm long, 4.41 ± 0.16 μm basal diameter, *n* = 45) with a thin socket, straight or slightly curved, longitudinally grooved, with sharp tip (Figures 2a,b,d,e and 3a–c,f). They were present on all segments and their number increased towards the tip of the antenna. There were three on the anterior edge of scape and six sc1 were in a line around the apical portion of segments at two‐thirds of the length from the pedicel (Figures 2a,b and 4d; Table 2) to the fourth flagellomere (Figures 2a,e and 3a–c). On the average 8 sc1 for each segment are present from fifth to eighth flagellomeres (Figures 2a,d,e and 3b) and 44–46 are on ninth flagellomere (Figure 4a,b).

**TABLE 2:** Mean numbers and distribution of sensilla on the antenna (*n* = 3) of *C. canaliculatum* males and females

				Flagellomeres								
Type	Sex	Scape	Pedicel	1	2	3	4	5	6	7	8	9
sc1	**♂**	3	6	6	6	6	6	8	8	8	8	44
	**♀**	3	6	6	6	6	6	8	8	8	8	46
sc2	**♂**	—	—	—	—	—	—	8	8	8	8	—
	**♀**	—	—	—	—	—	—	8	8	8	8	—
sb1	**♂**	—	—	—	—	—	—	30	46	47	50	150
	**♀**	—	—	—	—	—	—	30	36	49	49	150
sb2	**♂**	36	20	16	2	2	2	2	4	—	—	—
	**♀**	36	20	16	2	2	2	2	4	—	—	—
sco	**♂**	—	—	—	—	—	—	1	1	1	1	16
	**♀**							1	1	2	2	16
sca	**♂**	—	—	—	—	—	—	—	—	—	—	6
	**♀**	—	—	—	—	—	—	—	—	—	—	6

Sensilla chaetica type 2 (sc2) have smooth surface and straight bristle with blunt tip and are inserted tightly into a small cuticular socket (Figure 2a,d,e). Sc2 are 43 ± 2.55 μm in length and the basal diameter is 3.66 ± 0.21 μm (*n* = 4). They are distributed both ventrally and dorsally from fifth to eighth flagellomere in alternate position with sc1 (Figures 2a and 3c; Table 2).

#### 3.1.2 Sensilla basiconica

Sensilla basiconica type 1 (sb1) are pegs without a socket, curved in the distal direction of the antennal surface forming an angle of 80–90° (Figures 2d,e and 3b–d,f). They had a length of 13.25 ± 0.52 μm and a basal diameter 1.71 ± 0.06 μm (*n* = 22). On the average 30–50 sb1 were found clustered on the fifth, sixth, seventh, and eighth flagellomere to form a ventral sensorial area of 4,482 ± 551 μm^2^ (*n* = 4; Figures 2d,e and 3b–d,f). This type was also found on the ninth flagellomere in an apical sensory field where approximately 150 units were present (Figure 4a,b).

Sensilla basiconica type 2 (sb2) were very tiny cones with a sharp tip (Figures 2b,d and 3e). They had a length of 2.7 μm and a diameter at the base of 1.36 μm and were distributed in variable number from the scape (Figure 4d) to the sixth flagellomere (Table 2).

#### 3.1.3 Sensilla coeloconica

Sensilla coeloconica (sco) were small pit‐like organs located from fifth to eighth flagellomeres associated ventrally with the sb1. Sco were distributed one on each flagellomere in males (Figure 3b,c), while there were present in pair on the seventh and eighth flagellomeres of females (Figure 3d). There were 16 of them on the ninth flagellomere (Table 2), clustered on the anterior side of the tip (Figure 4a,c). The pit orifice (2.45 ± 0.04 μm in diameter, *n* = 6) was surrounded by a cuticular collar (9.97 ± 0.26 μm in diameter, *n* = 8; Figure 4c).

#### 3.1.4 Sensilla campaniformia

Sensilla campaniformia (sca) were small domes (approximately 1 μm in diameter) located at the apical tip of the ninth flagellomere (Figure 4b). They were placed in an apical ring of six units in both males and females (Table 2).

#### 3.1.5 Böhm sensilla

Böhm sensilla (bs) were short, smooth, sharp‐tipped, thorn‐like bristles located at the base (condyle) of the scape (Figure 4d). They had a length of 2.7 μm and a diameter at the base of 1.4 μm.

### 3.2 Glandular pores

In all specimens examined, pores were present on the antennomeres. They were usually associated with sb1 and sco from fifth to eighth flagellomeres in the ventral sensorial field (Figure 3c,d). Other glandular pores were located on ninth antennomere (Figure 4c). They were round or oval and measured about 500 nm. The distribution of the glandular pores showed no sexual dimorphism.

We also identified large areas of sensilla dorsally in the frontal and antennal grooves and ventrally on the mentum and gular grooves (Figure 1b–g).

## 4 DISCUSSION

This is the first study on antennal sense organs of males and females in *C. canaliculatum*. We identified and measured seven types of sensilla that show differences in morphology, abundance and, distribution on the antenna of *C. canaliculatum* as an adaptation to improve their efficiency and sensitivity in perceiving chemical and physical stimuli.

The most abundant type of sensilla was sc1 on the antenna and occurred in association with sc2 from fifth to eighth flagellomere. The wide articular socket, the pointed tip of the long bristles, and the longitudinal grooves as well as ultrastructural evidences in previously described species (Altner, Schaller‐Selzer, Stetter, & Wohlrab, 1983), suggested that the mechanoreception is the most likely function of sc1. In addition, mainly those present on scape and pedicel probably have a proprioceptive function, giving information about the position of antennae in relation to the body (Schneider, 1964). Sc2 without an articular socket and blunt‐tipped likely acts as a chemoreceptor (Altner & Prillinger, 1980). Sensilla, showing the external morphology of sc2 in *C. canaliculatum*, have already been described in other coleopteran species (Faucheux, Németh, & Kundrata, 2020; Hao et al., 2020; Seada & Hamza, 2018; Shao et al., 2019), including ground beetles, for example, *Paussus favieri* Fairmaire, 1851 (Di Giulio et al., 2012), *Bembidion lampros* Hdst (Merivee et al., 2002), even though in some case they are named as sensilla trichodea. In electrophysiological studies on *Pterostichus aethiops* (Panzer, 1796), a species that lives mainly in forest habitats, sc2 are referred to as contact chemoreceptors that can detect changes in salinity and pH (Merivee et al., 2004). The porous structures found mainly at the base of sb1 and sco in *C. canaliculatum* are part of glands associated with sensilla and are likely responsible for secretion of mucous‐like substances for lubrication of the antennal surface (Giglio, Ferrero, & Brandmayr, 2005).

Sensilla basiconica type 1 (sb1) are common on the antenna of several coleopteran species (Bartlet, Romani, Williams, & Isidoro, 1999; Faucheux, 2011; Faucheux, Hamidi, Mercadal, Thomas, & Frérot, 2019; Faucheux, Németh, & Kundrata, 2020; Jourdan, Barbier, Bernard, & Ferran, 1995; Lopes, Barata, Mustaparta, & Araújo, 2002; Romani et al., 2019) and they are known to be chemoreceptors (Altner & Prillinger, 1980; Keil & Steinbrecht, 1984; Zacharuk, 1985), as observed in *Nebria brevicollis* (Daly & Ryan, 1979). They are located on the antennal ventral site of *C. canaliculatum* from fifth to eighth and on ninth flagellomere in association with sco. Further ultrastructural analyses may be elucidate their possible function. Sco is a sensory peg located in a chamber connected to the environment by an opening and have been previously found in antennae of ants (Kleineidam, Romani, Tautz, & Isidoro, 2000; Ruchty et al., 2009), beetles (Giglio, Perrotta, Talarico, Zetto Brandmayr, & Ferrero, 2013; Merivee et al., 2002; Zauli et al., 2016a, 2016b), and katydids (Schneider, Kleineidam, Leitinger, & Römer, 2018). Ultrastructural and physiological analyses have shown that they function as thermo‐ and hygroreceptors (Altner, 1985; Ruchty et al., 2009; Schneider et al., 2018). In Drosophilidae, the involvement of sco in chemoreception has been studied for generalist and specialist species that have a shift in ecological niche (Nemeth, Ammagarahalli, Layne, & Rollmann, 2018). A carbon dioxide receptor has often been associated to thermoreceptor and hygroreceptor cells within this sensillum in species that have special requirements in the microclimatic detection of humidity, temperature, and CO_2_ concentration, as observed in fungus gardening leaf‐cutting ants (Kleineidam et al., 2000; Kleineidam & Tautz, 1996).

Sensilla basiconica type 2 were found from the scape to the sixth flagellomere of *C. canaliculatum*. Their morphology correspond to those of contact mechanoreceptors likely involved in the perception of antennal distortions caused by external stimuli (Giglio, Perrotta, & Zetto Brandmayr, 2010; Keil, 1997; Zacharuk, 1985).

Sensilla campaniformia (sca), described as no‐pore sensilla with a cupola or dome‐shaped external apparatus (Altner, 1977), have been found on mouthparts, antenna, wing bases, and legs to be involved in exteroception and proprioception (Chapman, 2012; Keil & Steinbrecht, 1984; Kim & Yamasaki, 1996; McIver, 1975, 1985; Schneider, 1964). Ultrastructural analyses of cerci in crickets indicated that they function as mechanoreceptors (Keil, 1997). The position on the antennal tip may allow it to detect environmental mechanical stimuli and regulate movement and locomotion of *C. canaliculatum* within the dead wood, replacing the scanning function of the eyes.

Böhm sensilla, typically present on the scape and pedicel in various insects, function to monitor antennal movements and position relatively to animal body and substrate (Faucheux, 2011; Faucheux et al., 2019; Faucheux, Németh, Hoffmannova, et al., 2020; Faucheux & Kundrata, 2017; Zacharuk, 1985; Zauli et al., 2016a, 2016b). Their location in the basal part of scape in *C. canaliculatum* suggests proprioception as a possible function.

Sensilla found on large areas of the head in *C. canaliculatum* have been previously described as olfactory receptors (Altner, 1985; Altner & Prillinger, 1980; Zacharuk, 1980) likely involved in prey choice or habitat location (Bartlet et al., 1999; Ren, Shi, Zhang, & Luo, 2012).

In Rhysodidae, morphology and distribution of antennal sensilla have so far been described only in males and females of *Omoglymmius americanus* (Baker, 2001). Sensilla trichodea, basiconica, coeloconica, and ampullacea have been identified. Sensilla trichodea, basiconica type 1 and ampullacea of *O. americanus* are morphological similar to sensilla chaetica, basiconica, and coeloconica, respectively, found in *C. canaliculatum*. However, it is difficult to compare the sensory patterns of both species because there is no univocal terminology for identifying sensilla from external morphology, and only ultrastructural and physiological analyses can delineate similarities or differences in the function. Moreover, distribution pattern, number and types of sensilla found in this species differ to *C. canaliculatum* because the evolutionary adaptation to different biotic and abiotic factors of its ecological niche.

## 5 CONCLUSION

This preliminary study described the sensory equipment for the first time in *C. canaliculatum*, a species belonging to Rhysodidae. We found no significant sexual differences in types, numbers, and distribution of sensilla in the antennae, except for the sensilla coeloconica. The distribution pattern of sensilla suggested that the antennae are involved in the scanning surrounding area for habitat selection to compensate for the absence of eyes (anophthalmic species). Furthermore, we assessed that the ability to detect temperature and humidity variation is crucial for identifying habitats where the amoeboid stage of Myxomycetes on which it feeds are present and for avoid overheating and dehydration. In addition, chemoreception can be useful in locating rotting trees suitable for laying eggs and providing food for their larval stages. Further ultrastructural and electrophysiological studies of antennae are needed to clarify our hypotheses on the functional role of these sensilla in the behavioral process of habitat selection, localization, and recognition. The results are a contribution to the knowledge and conservation of these species.

## CONFLICT OF INTEREST

The authors declare that they have no conflict of interests.

## AUTHOR CONTRIBUTIONS

Anita Giglio conceived research. Antonio Mazzei conduced field sampling. Maria Luigia Vommaro conducted scanning electron microscopy protocol, image processing and statistical analyses. Anita Giglio wrote the manuscript and secured funding. Pietro Brandmayr revised the final draft. All authors read and approved the manuscript.

## Data Availability

The data that support the findings of this study are available from the corresponding author upon reasonable request.

## References

[jemt23969-bib-0001] Abd El‐Ghany, N. M. , & Abd El‐Aziz, S. E. (2021). Morphology of antennae and mouthpart sensillae in *Lasioderma serricorne* (Fabricius) (Coleoptera: Anobiidae). Journal of Stored Products Research, 90, 101754. 10.1016/j.jspr.2020.101754

[jemt23969-bib-0002] Altner, H. (1977). Insect sensillum specificity and structure: An approach to a new typology. Olfaction and Taste, 6, 295–303.

[jemt23969-bib-0003] Altner, H. (1985). Ultrastructure and function of insect thermo‐ and hygroreceptors. Annual Review of Entomology, 30(1), 273–295. 10.1146/annurev.ento.30.1.273

[jemt23969-bib-0004] Altner, H. , & Prillinger, L. (1980). Ultrastructure of invertebrate chemo‐, thermo‐, and hygroreceptors and its functional significance. International Review of cytology, 67(Issue C), 69–139. 10.1016/S0074-7696(08)62427-4

[jemt23969-bib-0005] Altner, H. , Schaller‐Selzer, L. , Stetter, H. , & Wohlrab, I. (1983). Poreless sensilla with inflexible sockets. Cell and Tissue Research, 234(2), 279–307. 10.1007/bf00213769 6196120

[jemt23969-bib-0006] Baker, G. T. (2001). Morphology and distribution of sensilla on the antenna of *Omoglymmius americanus* (Laporte 1836) (Coleoptera: Rhysodidae). Proceedings of the Entomological Society of Washington, 103(1), 135–142. https://www.biodiversitylibrary.org/part/56644

[jemt23969-bib-0007] Bartlet, E. , Romani, R. , Williams, I. H. , & Isidoro, N. (1999). Functional anatomy of sensory structures on the antennae of *Psylliodes chrysocephala* L. (Coleoptera: Chrysomelidae). International Journal of Insect Morphology and Embryology, 28(4), 291–300. 10.1016/S0020-7322(99)00032-X

[jemt23969-bib-0008] Bell, R. T. (1994). Beetles that cannot bite: Functional morphology of the head of adult rhysodines (Coleoptera: Carabidae or Rhysodidae). The Canadian Entomologist, 126(3), 667–672.

[jemt23969-bib-0009] Bouget, C. , Larrieu, L. , & Brin, A. (2014). Key features for saproxylic beetle diversity derived from rapid habitat assessment in temperate forests. Ecological Indicators, 36, 656–664. 10.1016/j.ecolind.2013.09.031

[jemt23969-bib-0010] Bousquet, Y. , & Goulet, H. (1984). Notation of primary setae and pores on larvae of Carabidae (Coleoptera: Adephaga). Canadian Journal of Zoology, 62(4), 573–588. 10.1139/z84-085

[jemt23969-bib-0011] Carpaneto, G. M. , Baviera, C. , Biscaccianti, A. B. , Brandmayr, P. , Mazzei, A. , Mason, F. , … Audisio, P. (2015). A red list of Italian saproxylic beetles: Taxonomic overview, ecological features and conservation issues (Coleoptera). Fragmenta Entomologica, 47(2), 53. 10.4081/fe.2015.138

[jemt23969-bib-0012] Chapman, R. F. (2012). The insects: Structure and function. New York: Cambridge University Press.

[jemt23969-bib-0013] Chen, H. B. , Zhang, Z. , Wang, H. B. , & Kong, X. B. (2010). Antennal morphology and sensilla ultrastructure of *Dendroctonus valens* LeConte (Coleoptera: Curculionidae, Scolytinae), an invasive forest pest in China. Micron, 41(7), 735–741. 10.1016/j.micron.2010.06.007 20643555

[jemt23969-bib-0014] Daly, P. J. , & Ryan, M. F. (1979). Ultrastructure of antennal sensilla of *Nebria brevicollis* (Fab.) (Coleoptera: Carabidae). International Journal of Insect Morphology and Embryology, 8(3–4), 169–181. 10.1016/0020-7322(79)90015-1

[jemt23969-bib-0015] Di Giulio, A. , Maurizi, E. , Rossi Stacconi, M. V. , & Romani, R. (2012). Functional structure of antennal sensilla in the myrmecophilous beetle *Paussus favieri* (Coleoptera, Carabidae, Paussini). Micron, 43(6), 705–719. 10.1016/j.micron.2011.10.013 22365951

[jemt23969-bib-0016] Di Palma, A. , Pistillo, M. , Griffo, R. , Garonna, A. P. , & Germinara, G. S. (2019). Scanning electron microscopy of the antennal sensilla and their secretion analysis in adults of *Aromia bungii* (Faldermann, 1835) (Coleoptera, Cerambycidae). Insects, 10(4), 88. 10.3390/insects10040088 PMC6523298 30925753

[jemt23969-bib-0017] Dong, Z. , Yang, Y. , Dou, F. , Zhang, Y. , Huang, H. , Zheng, X. , … Lu, W. (2020). Observations on the ultrastructure of antennal sensilla of adult *Glenea cantor* (Cerambycidae: Lamiinae). Journal of Insect Science, 20(2), 1–9. 10.1093/jisesa/ieaa013 PMC7082121 32191795

[jemt23969-bib-0018] Elgar, M. A. , Zhang, D. , Wang, Q. , Wittwer, B. , Pham, H. T. , Johnson, T. L. , … Coquilleau, M. (2018). Insect antennal morphology: The evolution of diverse solutions to odorant perception. Yale Journal of Biology and Medicine, 91(4), 457–469. 30588211 PMC6302626

[jemt23969-bib-0019] Faucheux, M. J. (2011). Antennal sensilla of the yellow longicorn beetle *Phoracantha recurva* Newman, 1840: Distribution and comparison with *Phoracantha semipunctata* (Fabricius, 1775) (Coleoptera: Cerambycidae). Bulletin de l'Institut Scientifique, Rabat, Section Sciences de la Vie, 33(1), 19–29. http://www.israbat.ac.ma/IMG/pdf/BIS_SV_33_1_Faucheux.pdf

[jemt23969-bib-0020] Faucheux, M. J. (2013). Morphologie fonctionnelle des sensilles antennaires de *Scaurus gigas* Walt, 1835 (Coleoptera: Tenebrionidae). Bulletin de la Société des Sciences Naturelles de l'Ouest de la France, 35(2), 109–125.

[jemt23969-bib-0021] Faucheux, M. J. , Hamidi, R. , Mercadal, M. , Thomas, M. , & Frérot, B. (2019). Antennal sensilla of male and female of the nut weevil, *Curculio nucum* Linnaeus, 1758 (Coleoptera: Curculionidae). Annales de la Société Entomologique de France, 55(5), 395–409. 10.1080/00379271.2019.1649093

[jemt23969-bib-0022] Faucheux, M. J. , & Kundrata, R. (2017). Comparative antennal morphology of male Drilini with special reference to the sensilla (Coleoptera: Elateridae: Agrypninae). Zoologischer Anzeiger, 266, 105–119. 10.1016/j.jcz.2016.11.002

[jemt23969-bib-0023] Faucheux, M. J. , Németh, T. , Hoffmannova, J. , & Kundrata, R. (2020). Scanning electron microscopy reveals the antennal micromorphology of *Lamprodila (Palmar) festiva* (Coleoptera: Buprestidae), an invasive pest of ornamental Cupressaceae in Western Palaearctic. Biology, 9(11), 1–20. 10.3390/biology9110375 PMC7694220 33158061

[jemt23969-bib-0024] Faucheux, M. J. , Németh, T. , & Kundrata, R. (2020). Comparative antennal morphology of *Agriotes* (Coleoptera: Elateridae), with special reference to the typology and possible functions of sensilla. Insects, 11(2), 137. 10.3390/insects11020137 PMC7074560 32098184

[jemt23969-bib-0025] García, N. , Numa, C. , Bartolozzi, L. , Brustel, H. , Buse, J. , Norbiato, M. , … Galante, E. (2019). The conservation status and distribution of Mediterranean saproxylic beetles. Malaga, Spain: IUCN. 10.2305/iucn.ch.2018.ra.3.en

[jemt23969-bib-0026] Giglio, A. , Brandmayr, P. , Ferrero, E. A. , Giulianini, P. G. , Perrotta, E. , Talarico, F. F. , & Zetto Brandmayr, T. (2008). Ultrastructure of the antennal sensorial appendage of larvae of *Ophonus ardosiacus* (Lutshnik, 1922) (Coleoptera, Carabidae) and possible correlations between size and shape and the larval feeding habits. Zoologischer Anzeiger, 247(3), 209–221. 10.1016/j.jcz.2007.12.001

[jemt23969-bib-0027] Giglio, A. , Perrotta, E. , Talarico, F. , Zetto Brandmayr, T. , & Ferrero, E. A. (2013). Sensilla on maxillary and labial palps in a helicophagous ground beetle larva (Coleoptera, Carabidae). Acta Zoologica, 94(3), 324–330. 10.1111/j.1463-6395.2011.00558.x

[jemt23969-bib-0028] Giglio, A. , Ferrero, E. A. , Perrotta, E. , Talarico, F. F. , & Zetto Brandmayr, T. (2010). Sensory structures involved in prey detection on the labial palp of the ant‐hunting beetle *Siagona europaea* Dejean 1826 (Coleoptera, Carabidae). Acta Zoologica, 91(3), 328–334. 10.1111/j.1463-6395.2009.00414.x

[jemt23969-bib-0029] Giglio, A. , Ferrero, E. A. , & Brandmayr, T. Z. (2005). Ultrastructural identification of the antennal gland complement in *Siagona europaea* Dejean 1826, a myrmecophagous carabid beetle. Acta Zoologica, 86(3), 195–203. 10.1111/j.1463-6395.2005.00199.x

[jemt23969-bib-0030] Hammond, P. M. , & Lawrence, J. F. (1989). Mycophagy in insects: A summary. Insect‐Fungus Interactions, 14, 275–324. 10.1016/b978-0-12-751800-8.50018-5

[jemt23969-bib-0031] Hao, Y. N. , Sun, Y. X. , & Liu, C. Z. (2020). Functional morphology of antennae and sensilla of *Hippodamia variegata* (Coleoptera: Coccinellidae). PLoS ONE, 15(8 August), e0237452. 10.1371/journal.pone.0237452 32764805 PMC7413517

[jemt23969-bib-0032] Jonsell, M. , & Weslien, J. (2003). Felled or standing retained wood—It makes a difference for saproxylic beetles. Forest Ecology and Management, 175(1–3), 425–435. 10.1016/S0378-1127(02)00143-3

[jemt23969-bib-0033] Jourdan, H. , Barbier, R. , Bernard, J. , & Ferran, A. (1995). Antennal sensilla and sexual dimorphism of the adult ladybird beetle *Semiadalia undecimnotata* Schn. (Coleoptera: Coccinellidae). International Journal of Insect Morphology and Embryology, 24(3), 307–322. 10.1016/0020-7322(95)98584-Z

[jemt23969-bib-0034] Keil, T. A. (1997). Functional morphology of insect mechanoreceptors. Microscopy Research and Technique, 39(6), 506–531. 10.1002/(SICI)1097-0029(19971215)39:6<506::AID-JEMT5>3.0.CO;2-B 9438251

[jemt23969-bib-0035] Keil, T. A. , & Steinbrecht, R. A. (1984). Mechanosensitive and olfactory sensilla of insects. In King, R. C. , & Akai, H. (eds) Insect ultrastructure (pp. 477–516). Boston, MA: Springer. 10.1007/978-1-4613-2715-8_13

[jemt23969-bib-0036] Kim, J. L. , & Yamasaki, T. (1996). Sensilla of *Carabus (Isiocarabus) fiduciarius saishutoicus* Csiki (Coleoptera: Carabidae). International Journal of Insect Morphology and Embryology, 25(1–2), 153–172.

[jemt23969-bib-0037] Kleineidam, C. , & Tautz, J. (1996). Perception of carbon dioxide and other “air‐condition” parameters in the leaf cutting ant *Atta cephalotes* . Naturwissenschaften, 83(12), 566–568. 10.1007/bf01141981

[jemt23969-bib-0038] Kleineidam, C. , Romani, R. , Tautz, J. , & Isidoro, N. (2000). Ultrastructure and physiology of the CO2 sensitive sensillum ampullaceum in the leaf‐cutting ant *Atta sexdens* . Arthropod Structure and Development, 29(1), 43–55. 10.1016/S1467-8039(00)00012-8 18088913

[jemt23969-bib-0039] Lopes, O. , Barata, E. N. , Mustaparta, H. , & Araújo, J. (2002). Fine structure of antennal sensilla basiconica and their detection of plant volatiles in the eucalyptus woodborer, *Phoracantha semipunctata* Fabricius (Coleoptera: Cerambycidae). Arthropod Structure and Development, 31(1), 1–13. 10.1016/S1467-8039(02)00011-7 18088966

[jemt23969-bib-0040] Makarov, K. V. (2008). Towards a new synthesis amongst taxonomic, ecological and biogeographical approaches in carabidology. In Back to the roots and back to the future (Vol. 75, pp. 101–124). Sofia‐Moscow: Pensoft Publishers.

[jemt23969-bib-0041] Mazzei, A. , Audisio, P. , Taglianti, A. V. , & Brandmayr, P. (2019). Geographical distribution and conservation status of the threatened saproxylic beetles *Rhysodes sulcatus* (Fabricius, 1787), *Clinidium canaliculatum* (O.G. Costa, 1839) and *Omoglymmius germari* (Ganglbauer, 1891) in Italy (Coleoptera: Rhysodidae). Fragmenta Entomologica, 51(1), 89–96. 10.4081/fe.2019.337

[jemt23969-bib-0042] McIver, S. B. (1975). Structure of cuticular mechanoreceptors of arthropods. Annual Review of Entomology, 20(1), 381–397. 10.1146/annurev.en.20.010175.002121 1090241

[jemt23969-bib-0043] McIver, S. B. (1985). Mechanoreception. In G. A. Kerkut & L. I. Gilbert (Eds.), Comparative insect physiology, biochemistry and pharmacology (pp. 71–132). New York: Pergamon Press.

[jemt23969-bib-0044] Merivee, E. , Ploomi, A. , Rahi, M. , Bresciani, J. , Ravn, H. P. , Luik, A. , & Sammelselg, V. (2002). Antennal sensilla of the ground beetle *Bembidion properans* Steph. (Coleoptera, Carabidae). Micron, 33(5), 429–440. 10.1016/S0968-4328(02)00003-3 11976030

[jemt23969-bib-0045] Merivee, E. , Renou, M. , Mänd, M. , Luik, A. , Heidemaa, M. , & Ploomi, A. (2004). Electrophysiological responses to salts from antennal chaetoid taste sensilla of the ground beetle *Pterostichus aethiops* . Journal of Insect Physiology, 50(11), 1001–1013. 10.1016/j.jinsphys.2004.09.001 15607503

[jemt23969-bib-0046] Nemeth, D. C. , Ammagarahalli, B. , Layne, J. E. , & Rollmann, S. M. (2018). Evolution of coeloconic sensilla in the peripheral olfactory system of *Drosophila mojavensis* . Journal of Insect Physiology, 110, 13–22. 10.1016/j.jinsphys.2018.08.003 30107159

[jemt23969-bib-0047] Ploomi, A. , Merivee, E. , Rahi, M. , Bresciani, J. , Ravn, H. P. , Luik, A. , & Sammelselg, V. (2003). Antennal sensilla in ground beetles (Coleoptera, Carabidae). Agronomy Research, 1(2), 221–228.

[jemt23969-bib-0048] Ren, L. , Shi, J. , Zhang, Y. , & Luo, Y. (2012). Antennal morphology and sensillar ultrastructure of *Dastarcus helophoroides* (Fairmaire)(Coleoptera: Bothrideridae). Micron, 43(9), 921–928. 22522119 10.1016/j.micron.2012.03.005

[jemt23969-bib-0049] Romani, R. , Bedini, S. , Salerno, G. , Ascrizzi, R. , Flamini, G. , Echeverria, M. C. , … Conti, B. (2019). Andean flora as a source of new repellents against insect pests: Behavioral, morphological and electrophysiological studies on *Sitophilus zeamais* (coleoptera: Curculionidae). Insects, 10(6), 171. 10.3390/insects10060171 PMC6628024 31207971

[jemt23969-bib-0050] Ruchty, M. , Romani, R. , Kuebler, L. S. , Ruschioni, S. , Roces, F. , Isidoro, N. , & Kleineidam, C. J. (2009). The thermo‐sensitive sensilla coeloconica of leaf‐cutting ants (*Atta vollenweideri*). Arthropod Structure and Development, 38(3), 195–205. 10.1016/j.asd.2008.11.001 19095080

[jemt23969-bib-0051] Schneider, D. (1964). Insect antennae. Annual Review of Entomology, 9(1), 103–122. 10.1146/annurev.en.09.010164.000535

[jemt23969-bib-0052] Schneider, E. S. , Kleineidam, C. J. , Leitinger, G. , & Römer, H. (2018). Ultrastructure and electrophysiology of thermosensitive sensilla coeloconica in a tropical katydid of the genus *Mecopoda* (Orthoptera, Tettigoniidae). Arthropod Structure and Development, 47(5), 482–497. 10.1016/j.asd.2018.08.002 30120986

[jemt23969-bib-0053] Seada, M. A. , & Hamza, A. M. (2018). Differential morphology of the sensory sensilla of antennae, palpi, foretarsi and ovipositor of adult *Tribolium castaneum* (Herbst) (Coleoptera:Tenebrionidae). Annals of Agricultural Sciences, 63(1), 1–8. 10.1016/j.aoas.2018.02.001

[jemt23969-bib-0054] Sevarika, M. , Rondoni, G. , Conti, E. , & Romani, R. (2021). Antennal sensory organs and glands of the harlequin ladybird, *Harmonia axyridis* . Entomologia Experimentalis et Applicata, 169(1), 111–124. 10.1111/eea.12948

[jemt23969-bib-0055] Shao, K. M. , Sun, Y. , Wang, W. K. , & Chen, L. (2019). A SEM study of antennal sensilla in *Maladera orientalis* Motschulsky (Coleoptera: Scarabaeidae: Melolonthinae). Micron, 119, 17–23. 10.1016/j.micron.2019.01.004 30639944

[jemt23969-bib-0056] Shi, X. , Zhang, S. F. , Liu, F. , Zhang, Z. , Xu, F. Y. , Yin, S. Y. , & Kong, X. B. (2021). Sensilla on antennae and mouthparts of adult spruce bark beetle *Ips typographus* (Coleoptera: Curculionidae). Microscopy Research and Technique. 84, 1484–1497. 10.1002/jemt.23704 33470484

[jemt23969-bib-0057] Stokland, J. N. , Siitonen, J. , & Jonsson, B. G. (2012). Biodiversity in dead wood. New York: Cambridge University Press. 10.1017/CBO9781139025843

[jemt23969-bib-0058] Wang, H. , Zheng, H. , Zhang, Y. , & Zhang, X. (2018). Morphology and distribution of antennal, maxillary palp and labial palp sensilla of the adult bruchid beetles, *Callosobruchus chinensis* (L.) (Coleoptera: Bruchidae). Entomological Research, 48(6), 466–479. 10.1111/1748-5967.12296

[jemt23969-bib-0059] Zacharuk, R. Y. (1980). Ultrastructure and function of insect chemosensilla. Annual Review of Entomology, 25, 27–47. 10.1146/annurev.en.25.010180.000331

[jemt23969-bib-0060] Zacharuk, R. Y. (1985). Antennae and sensilla. In Comprehensive insect physiology, biochemistry and pharmacology nervous system: Sensory (Vol. 6, pp. 1–69). Oxford: Pergamon Press.

[jemt23969-bib-0061] Zauli, A. , Maurizi, E. , Carpaneto, G. M. , Chiari, S. , Merivee, E. , Svensson, G. P. , & Di Giulio, A. (2016a). Scanning electron microscopy analysis of the antennal sensilla in the rare saproxylic beetle *Elater ferrugineus* (Coleoptera: Elateridae). Italian Journal of Zoology, 83(3), 338–350. 10.1080/11250003.2016.1211766

[jemt23969-bib-0062] Zauli, A. , Maurizi, E. , Carpaneto, G. M. , Chiari, S. , Svensson, G. P. , & Di Giulio, A. (2016b). Antennal fine morphology of the threatened beetle *Osmoderma eremita* (Coleoptera: Scarabaeidae), revealed by scanning electron microscopy. Microscopy Research and Technique, 79(3), 178–191. 10.1002/jemt.22618 26789276

